# Anaesthesia Management of a Pregnant Woman with Glutaric Aciduria Type 1 Undergoing Cesarean Section

**DOI:** 10.4274/TJAR.2025.241705

**Published:** 2025-02-11

**Authors:** Yağmur Demirel, Sevgi Kesici, Celal Kaya, Sibel Oba, Hacer Şebnem Türk

**Affiliations:** 1 University of Health Sciences Türkiye Şişli Hamidiye Etfal Training and Research Hospital, Clinic of Anaesthesiology and Reanimation, İstanbul, Türkiye

**Keywords:** Cesarean section, glutaric aciduria type 1, neuraxial anaesthesia, pregnancy

## Abstract

Glutaric aciduria type 1 (GA-1) presents unique challenges for anaesthetists. This case report discusses anaesthesia management in a pregnant woman with GA-1 undergoing cesarean delivery. Based on a cautious consideration of potential complications, combined spinal-epidural anaesthesia was preferred in this case. Maintenance of normoglycemia, normothermia, low-protein diet, carnitine supplementation, and proper hydration were prioritized. A healthy baby was delivered without complications. This case underscores the importance of comprehensive preoperative assessment and individualized anaesthesia strategies for achieving optimal outcomes in pregnant patients with GA-1. The cautious management of anaesthesia-related risks is important to ensure patient safety and decrease stress responses. Neuraxial anaesthesia and analgesia may be advantageous in specific cases.

**Main Points**

• Glutaric aciduria type 1 is a rare metabolic disorder that poses unique challenges, particularly during the perioperative period.

• Cautious preoperative evaluation and preparation are important for reducing catabolic stress.

• As physiological changes during pregnancy pose unique challenges for anaesthesiologists, appropriate anaesthesia techniques and complication management techniques should be selected.

• Combined spinal-epidural anaesthesia can be used in selected pregnant patients to provide effective pain management while avoiding general anaesthesia and reducing catabolic stress.

## Introduction

Glutaric aciduria type 1 (GA-1) is a significant metabolic disorder caused by genetic deficiency in glutaryl-CoA dehydrogenase. Patients with GA-1 may exhibit diverse clinical features, particularly neurological disorders attributed to the accumulation of amino acids and other metabolites. Symptoms can manifest from childhood to adulthood.^1, 2^

Formation of non-toxic glutarylcarnitine via conjugation of glutaryl-CoA with carnitine is a physiological detoxification mechanism for glutaryl excretion and intracellular CoA regeneration. Nevertheless, this process can lead to secondary plasma carnitine deficiency, potentially contributing to complications such as cardiomyopathy, muscle weakness, and fatigue. Special attention must be given to patients with fever, infection, surgery, and pregnancy because they can present with acute encephalopathic crises and metabolic acidosis.^1, 2, 3^

Herein, we present the anaesthesia management of a pregnant woman diagnosed with GA-1 who underwent cesarean delivery.

## Case Presentation

A 30-year-old woman, with a height of 166 cm and weight of 82 kg, at 39 weeks of gestation and a history of GA-1 was admitted to the hospital. She had previously delivered via emergency cesarean section with regional anaesthesia, and the procedure had gone smoothly. The patient’s second pregnancy planned as elective cesarean section was reviewed preoperatively. Risks and anaesthesia methods were determined.

Preoperative echocardiography revealed normal functions. Further, the patient’s neurological examination result was normal, and previous magnetic resonance imaging revealed intracranial demyelinating plaques. After consultation with neurology, no contraindications for regional anaesthesia were identified. Additionally, a dietician was referred to prevent the catabolic process.

Combined spinal-epidural (CSE) anaesthesia and preparations for possible conversion to general anaesthesia were reviewed. Considering the risk of malignant hyperthermia, dantrolene was kept on standby. In the preoperative period, oral and intravenous carnitine treatments were switched. The risk of metabolic acidosis and ketonuria was evaluated by arterial blood gas analysis and complete urinalysis. Dextrose fluids and balanced solutions were preferred for perioperative fluid replacement. To avoid prolonged fasting, she was scheduled as the first case of the day.

CSE was performed at the lumbar 3^rd^-4^th^ interspace using a needle-through-needle technique with an 18-G Tuohy needle and a 27-G pencil-point spinal needle (Espocan® CSE set). After obtaining cerebrospinal fluid, 2 mL of 0.5% heavy bupivacaine was injected. The dosage was determined based on a regimen adjusted for height and weight, as used in our department. After the withdrawal of the spinal needle, the epidural catheter was advanced 5 cm into the epidural space. The patient was then positioned supine. A T4-level sensory block, confirmed using the pinprick method, was achieved within 8 min. The surgical duration was 90 minutes. During the perioperative period, the vital signs were as follows: heart rate, 65-105 beats min^-1^; mean blood pressure, 72-88 mmHg; and oxygen saturation level, 98-100%. Forced air warmers were used with frequent tympanic temperature measurements to achieve normothermia.

The patient delivered a baby boy weighing 2,300 g, with a normal physical examination. Following delivery, patient-controlled analgesia (PCA) was initiated using a mixture of bupivacaine and fentanyl after administering a test dose of 40 mg of lidocaine. The PCA mixture was prepared with a concentration of 2 µg mL^-1^ fentanyl and 1 mg mL^-1^ bupivacaine. The solution was administered at a bolus dose of 5 mL with a lockout interval of 15 min. The patient did not present with any pain during or after surgery. The surgical procedure was uneventful, and the patient was subsequently transferred to the intensive care unit. Urinalysis, arterial blood gas, lactate, and electrolytes were normal. The patient was closely monitored in the intensive care unit for 24 hours and was discharged without complications after 2 days.

## Discussion

GA-1 is a mitochondrial metabolic disorder in which minimizing catabolic stress is essential. Infections, stress, fever, pregnancy, or surgery can trigger encephalopathic crises, causing central nervous system (CNS) damage.^1, 2^

This case highlights the importance of comprehensive preoperative assessment and preparation. This includes evaluating respiratory and cardiac function and neurological status and reviewing previous medical records to identify potential anaesthesia risks.

There are no specific radiological findings in patients with GA-1, but CNS imaging may reveal suggestive clinical features, including hydrocephalus, brain atrophy, structural changes of the basal ganglia, and demyelination. However, non-traumatic subdural hematoma was also reported.^4^ Hence, conducting a comprehensive neurological examination prior to any anaesthetic procedure, seeking neurological consultation, and reviewing radiological images are important as they may help identify contraindications to regional anaesthesia.

Because the CNS is primarily affected, patients frequently experience dystonic movements and seizures. Thus, anticonvulsants should be readily available as per the neurologist’s recommendations, and routine anticonvulsants should be administered until the morning of surgery. Valproate can significantly disrupt the mitochondrial acetyl-CoA/CoA ratio and should be avoided.^2^

Patients with GA-1 are typically managed with a low-protein diet and carnitine and riboflavin supplementation. Considering the physiological changes caused by pregnancy, patients should be evaluated by a dietician immediately after hospital admission. Anaesthetists must ensure that intravenous dextrose fluids are administered preoperatively because fasting can cause protein degradation, leading to increased amino acid levels and clinical symptom onset. Switching from oral to intravenous carnitine supplementation can prevent secondary carnitine deficiency. In addition, decreasing fasting times and scheduling the surgery as the first surgery performed on the day reduces the risk of metabolic decompensation.^2, 3^

A rapid rise in plasma lactate levels has been observed after administering Ringer’s lactate to patients with mitochondrial disorders.^5^ This phenomenon is likely due to impaired lactate metabolism, suggesting that intravenous fluids containing lactate should generally be avoided.^6^ Similarly, we avoided Ringer’s lactate in our patient to prevent lactate accumulation.

General anaesthesia might be challenging because of concerns about neuromuscular blockers, inhalational anaesthesia, and propofol-related complications. Although low-dose propofol has been reported in the literature, long-term and high-dose propofol can inhibit mitochondrial electron transport.^7^ Patients may also have increased sensitivity and a prolonged response to neuromuscular blockers as neurological involvement in this disease.^8^ Another concern is the relationship between mitochondrial disease and malignant hyperthermia.^9^ Therefore, regional anaesthesia was preferred in our patient given that our evaluation did not show any contraindications.

To the best of our knowledge, this is the first report of CSE in a patient with GA-1. Studies have indicated that epidural anaesthesia alone does not provide sufficient analgesia for pregnant women undergoing surgical procedures, particularly in the sacral region.^10^ This can result in inadequate pain control during the perioperative period. Additionally, the slower onset of blockade with epidural anaesthesia compared with spinal anaesthesia poses a significant drawback in emergencies requiring rapid intervention.^11^ The CSE anaesthesia technique effectively addresses these challenges by combining rapid-onset spinal anaesthesia with the prolonged analgesic effects of epidural anaesthesia. This method not only ensures effective anaesthesia during surgery but also enables postoperative pain control via the epidural catheter. Furthermore, some studies have indicated that CSE anaesthesia enhances maternal hemodynamic stability and reduces the risk of hypotension.^12^ In our case, CSE anaesthesia provided a painless and comfortable experience during and after the procedure, minimizing the stress response. This approach also allowed for safe anaesthesia management by avoiding the potential adverse effects associated with intravenous or inhaled anaesthesia.

In patients with GA-1, in addition to pain management, treatment primarily aims to maintain normoglycemia and normothermia, implement a low-protein diet, prevent secondary depletion via carnitine supplementation, and ensure adequate hydration, electrolyte balance, and normal pH levels.

## Conclusion

In pregnant women with GA-1, preventing stress response via cautious anaesthesia management is important. Neuraxial anaesthesia and analgesia can achieve optimal outcomes in selected patients.

## Ethics

**Informed Consent:** The patient provided consent for the clinical information pertaining to the case to be published in a medical journal.
